# Removal of Fluorescein Dye from Aqueous Solutions Using Natural and Chemically Treated Pine Sawdust

**DOI:** 10.1155/2020/8824368

**Published:** 2020-11-25

**Authors:** Nada S. Al-Kadhi

**Affiliations:** Department of Chemistry, College of Science, Princess Nourah Bint Abdulrahman University, Riyadh, Saudi Arabia

## Abstract

The various factors affecting the removal of fluorescein dye using sawdust from aqueous solutions such as time, initial concentration, pH, and temperature were studied. The optimal conditions for removing the FD are 1 g of sawdust at pH 3 and 120 min time of contact. Dye removal dropped from 93.42% to 80.04% with natural pine sawdust (NPS) and from 96.83% to 81.51% with synthetic pine sawdust (SPS) by increasing their concentration from 2 to 10 mg/L. Isotherm, kinetic, and thermodynamic models were applied for determining their constants. The results indicated that the FD removal equilibrium was effectively defined by the Langmuir, Freundlich, and Temkin models. Kinetic studies showed that the pseudo-second order was well suited for dye removal, and the internal diffusion process was by two steps. The thermodynamic parameter values suggested that FD removal were physical adsorption, exothermic, lower randomness, and spontaneous.

## 1. Introduction

Currently, all communities are suffering from water pollution problems due to the discharge of industrial effluents to surface water from a wide range of industries such as textiles, leather, paper, printing, food, cosmetics, painting, rubber and plastics, pesticides and chemicals in the wood conservation and pharmaceutical industries [1–3]. The traces of the dyes are discharged without treatment into the aqueous media. The presence of these dyes in surface water has a deterrent effect on sunlight penetration and water field ventilation, thus reducing photosynthesis activity. Several methods are available for the treatment of dyes such as chemical oxidation experiments, flotation, adsorption, electrolysis, chemical coagulation, and biodegradation [4]. Adsorption allows measurements of kinetic and equilibrium, so it is an effective method for removing dyes from aqueous solutions.

Adsorbent materials have been used to treat aqueous solutions to remove different substances such as pigments, mineral ions, and other organic materials. These materials include perlite, activated carbon, bentonite, silica gels, fly ash, lignite, peat, silica, clay, tree roots, wood, teak powder and teak bark that have been evaluated for adsorption studies [5].

**Figure 1:**
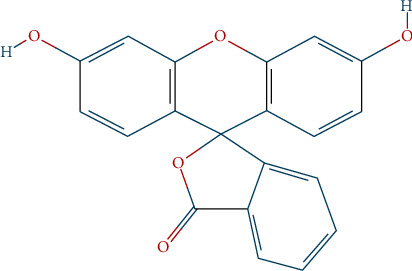
Chemical structure of the fluorescein (C_20_H_12_O_5_) dye.

Fluorescein, one of the commercially available textile dyes, has the following chemical structure in Figure 1:

Fluorescein, an organic compound with molecular formula C_20_H_12_O_5_, is commonly used as an artificial coloring agent. It is prepared by heating the resorcinol and phthalic anhydride on a zinc catalyst at a melting point between 314°C and 316°C, crystallizing as a dark red powder. Due to the strong green luster that gives alkaline solutions a visible color even in diluted solutions, fluorescein has been identified. It was used in analytical instruments, cosmetics, and as an aqueous detector for liquid coloring.

Sawdust is an active material used to eliminate various pollutants. The use of wood waste would reduce the total environmental waste [6, 7]. Sawdust, a relatively abundant and inexpensive material, has been extensively investigated as an adsorbent for removing contaminants from water [8].

Many agricultural residues, such as wheat straw, rice husk, corncobs, and wood chips, have been used successfully to adsorb individual dyes and dye mixtures in textile effluents [9]. Removal of methylene blue (MB) and other basic dyes has been carried out using beech sawdust [10, 11], wheat straw [12], cedar sawdust [13], rubberwood sawdust [14], kudzu [15], banana and orange peels [16] and palm kernel fiber [17].

Acid and alkali pretreated lignocellulosic materials (wheat straw, corncobs, barley husks, wood sawdust) were successfully used as adsorbents for a variety of dyes [18–20]. Beech sawdust [10, 11] has proven to be effective for basic dye adsorption in batch and fixed-bed systems.

A continuous procedure is to look for suitable and inexpensive natural materials. The purpose of this study is therefore to examine the use of natural and synthetic pine sawdust to eliminate fluorescein dye from aqueous solutions.

## 2. Materials

The fluorescein dye purchased from Sigma–Aldrich (St. Louis, MO, USA). The fluorescein dye solution was prepared with distilled water. Pinewood is the cheapest natural wood as Swedish wood and yellow wormwood, imported from Sweden or Turkey. Pine sawdust (NPS) was purchased from Saudi Arabia furniture factories.

### 2.1. Preparation of the Adsorbents

The NPS and SPS used were purchased from the carpentry workshop. The sawdust was ground to powder using the planetary ball mill (DECO-PBM-V-0.4L). An Octagon D200 Digital Sieve Shaker was used to sieve the powder into particles less than 200 *µ*m. The adsorbent materials were stored for reuse in glass bottles without prior treatment.

### 2.2. Preparation of FD Solution

The FD stock solution (1000 mg/L) was prepared with double distilled water. All solutions used in the experiments were prepared by dilution of the stock solution to a predefined concentration.

## 3. Methods

The adsorption experiments were performed in a series of flasks containing 100 ml dye solution at a specific concentration and adsorbent mass. For adsorption equilibrium, different concentrations of dye ranging from 2 to 10 mg/L were investigated. Furthermore, kinetics experiments were performed using 10 mg/L dye and 1 g of adsorbent at 25°C. A Shaker (“Rotaterm” orbital and linear shaker) was used to mix dyes and adsorbent material for 3 hours at 90 rpm. Furthermore, the contact time varied from 30 to 180 min. The initial concentration of 10 mg/L was used to study the effect of pH value (pH 3–11), contact time (30–180 min), and temperature (25–60°C) on 1 g of the adsorbed dye [21]. All the mixtures were filtered and the dye concentrations were measured using a 480 nm wavelength UV/V in a spectrophotometer (UV–V analysis was done by ultraviolet spectrum -type V-770 UV-Visible/NIR spectrophotometer-over a wavelength range of 200 to 800 nm). The dye adsorbed quantity (*q*_*e*_ (mg/g)) was determined at equilibrium by the following equation [21]:

(1) $$\begin{array}{c} q_{e}\;=\;\frac{\left(C_{0}\;-\;C_{e}\right)\;V}{m}, \end{array}$$

where *C*_0_ and *C*_*e*_ (mg/L) were the initial dye concentration and equilibrium concentration, respectively. V is the solution volume (*L*) and *m* is the adsorbent mass (g). The percentage of dye adsorption (% *R*_FD_) from the solution was determined as follows [7]:

(2) $$\begin{array}{c} \%\;R_{\text{FD}}\;=\;\frac{\left(C_{0}\;-\;C_{e}\right)}{C_{0}}\;\times 100.\; \end{array}$$

Adsorption kinetic models were developed to explain the adsorption behavior. Langmuir and Freundlich's isotherm models were used to study adsorption data. The processes of adsorption are described by calculating thermodynamic parameters, namely, enthalpy (∆*H*°), entropy (∆*S*°), and free energy (∆*G*°).

Scanning electron microscopy SEM was performed using the JSM-6380 LA scanning electron microscope for the adsorbent layer with a high resolution of 3.0 nm.

## 4. Results and Discussion

### 4.1. Characterization of the Adsorbents

**Figure 2:**
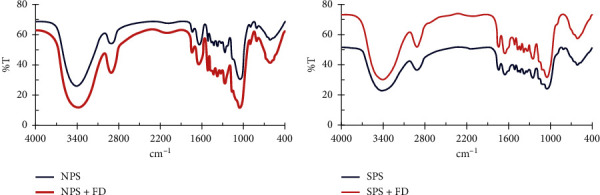
FT-IR analysis of the functional groups in (a) NPS and (b) SPS before and after the adsorption of FD.

Figures 2(a) and 2(b) describe the functional groups in the NPS and the SPS, respectively.  Figure 2(a) shows one broad peak at 3423.84 cm^−1^, which may be due to the hydroxyl group (phenolic and alcoholic). A strong peak was observed at 2900 cm^−1^, which is correlated with an Sp^3^ C-H stretching. Two peaks appear at 1730 and 1650 cm^−1^ for the C=O and C=C, respectively. Medium peaks appeared at 1450 and 1350 cm^−1^, which is attributed to Sp^2^ C-H and Sp^3^ C-H bending, respectively. The results of the FT-IR (Table 1) study of sawdust showed the presence of functional groups such as -OH (alcohol and carboxylic acid), CH (alkanes), C=C (alkalene), and C=O (carbonyl) found in NPS and SPS.  Figure 2(b) may be involved in removing dye from aqueous solutions [6, 22, 23].

### 4.2. FESEM Analysis

**Figure 3:**
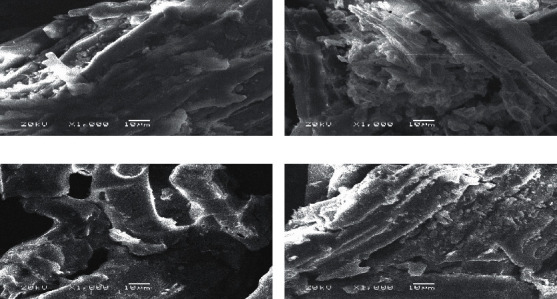
SEM photograph for surface of (a) NPS. (b) SPS before and after adsorption of FD.

Figures 3(a) and 3(b) show the morphological structure of NPS and SPS by using field emission scanning electron microscopy. The sawdust micrographs reveal the pores, which help greatly to absorb large quantities of FD molecules on the adsorbent surface. [6, 23–25]. Figures 3(a) and 3(b) indicate that FD covers the pores and that the pores are no longer visible. This is evidence of FD adsorption on the adsorbent material surface [26].

### 4.3. Effect of FD Concentration

**Figure 4:**
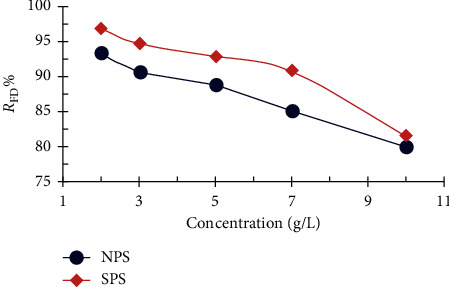
Effect of the initial concentration on the quantity of FD removal by NPS and SPS (1 g adsorbent dosage; 50 ml solution volume; 3 h contact time; and 25°C temperature).

Figure 4 explains the effect of the initial concentration on the percentage of removal of the dye by using both NPS and SPS. The removal percentage of the dye decreases from 97 to 80%, with an increased FD concentration from 2 to 10 mg/L due to low concentration driving force and sawdust saturation with the dye [27, 28]; for higher dye concentrations, active sites are occupied, and the number of active sites available for adsorption is decreased. Unabsorbed dye concentration caused lower *R*_FD_%.

### 4.4. Effect of Adsorbent Dose

**Figure 5:**
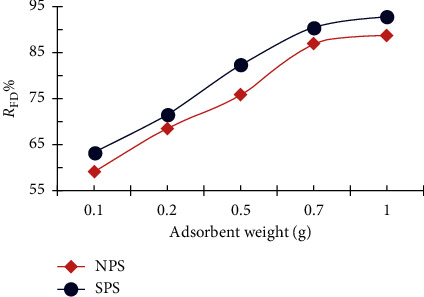
Adsorbent dose impact of NPS and SPS amounts on the FD removal (10 mg/L FD concentration; 50 ml solution volume; 3 h contact time; and 25°C temperature).

The effect of dose on FD removal was studied.  Figure 5 indicates a sharp increase in the percentage of FD removal with increased dose of SPS and NPS, reaching 90 and 85%, respectively, up to 0.7 g, due to increased availability of exchange sites and surface area with the increased amount of the adsorbate. It was also noted that the rate of increase in the rate of removal of the dye decreased and reached equilibrium after the dose of 0.7 g, probably due to the agglomeration of sawdust where the particular surface area and active sites are not significantly increased [29, 30].

### 4.5. Effect of Contact Time

**Figure 6:**
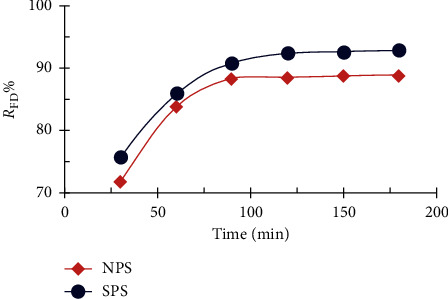
Contact time effect on NPS and SPS quantities of FD removal (1 g adsorbent dosage; 10 mg/L FD concentration; 50 ml solution volume; and 25°C temperature).

The effect of contact time on FD removal using NPS and SPS shows that the percentage of the removal of dye increased from 71.82 to 88.84% with NPS and from 75.80 to 92.93% with SPS (Figure 6), where the rate of dye removal increased rapidly during the initial stages to 90 min due to the presence of excessive porous active sites on the surface of the sawdust. Subsequently, the dye molecules saturated the surface of the sawdust, making it difficult to remove excess dye from the aqueous solution [24, 31, 32] while the Indian Rosewood sawdust adsorbs the methylene blue dye within 30 minutes, depending on the type of wood and the type of dye.

### 4.6. Effect of pH

**Figure 7:**
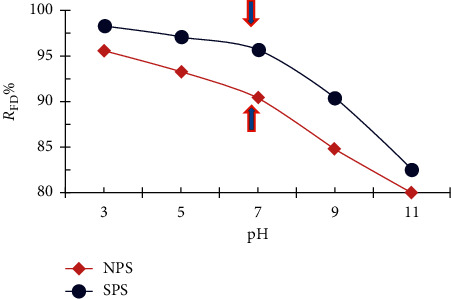
Percentage of removal of FD as a function of the pH solution (1 g adsorbent dosage; 10 mg/L FD concentration; 50 ml solution volume; and 25°C temperature).

Experiments were performed at different pH values between 3 and 11 in order to remove FD from the aqueous solution by sawdust.  Figure 7 shows a slight decrease in the percentage of FD removal by increasing the pH from 3 to 7 while at pH above 7, it is rapidly decreased. This is due to the FD being able to form cationic molecules, so both NPS and SPS tend to removing FD in an acidic medium more than in an alkaline medium [19, 33, 34]. These results are consistent with the malachite green adsorption by sulfuric acid treated sawdust carbon (SDC) and formaldehyde treated sawdust (SD). On the other hand, pH effect on methylene blue adsorption on beech sawdust gives opposite results [35].

### 4.7. Effect of Temperature

**Figure 8:**
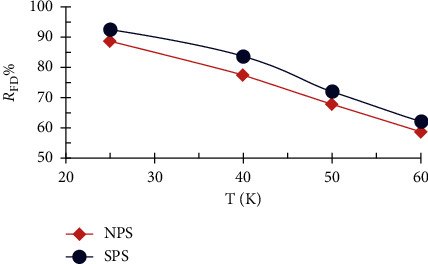
Effect of temperature on the percentage of FD removal by NPS and SPS (1 g adsorbent dosage; 10 mg/L concentration of FD; 50 ml solution volume; and 3 h contact time).

The results showed that the temperature ranging from 25 to 60°C plays an important role in the rate of FD removal by sawdust as shown in Figure 8. The percentage of FD removal decreased by increasing the temperature of the dye solution. The results show that the removal of the FD by sawdust is an exothermic process and the form of bond between the FD molecules and the active sites on the surface of the sawdust is a physical bond, such that bond breakage occurs at high temperature [36].

### 4.8. Adsorption Isotherms Models

The isotherm models were used to understand the of adsorption mechanism by using sawdust to remove FD from an aqueous solution. In accordance with the following equations, the Langmuir, Freundlich and Temkin isotherm models were used [5]:

(3) $$\begin{array}{c} \frac{C_{e}}{q_{e}}\;=\;\frac{1}{K_{L}q_{m}}+\frac{C_{e}}{q_{m}},\;\\ \log\;q_{e}\;=\;\log\;K_{F}+\frac{1}{n}\;\log\;C_{e},\\ q_{e}\;=\;B\;\ln\;A+B\;\ln\;C_{e}, \end{array}$$

where *C*_*e*_ = the adsorbate equilibrium concentration (mg/L), *q*_*e*_ = the observed adsorption capacity at equilibrium (mg/g), *q*_*m*_ = maximum absorption capacity (mg/g), *K*_*L*_ = the Langmuir constant (L/mg), *K*_*F*_ = the Freundlich constant (mg^(1−1/n)^ g^−1^L^1/n^), *n* = Freundlich equilibrium coefficient, *B* = the Temkin constant (J/mol), and *A* = the equilibrium binding constant (L/mg).

**Figure 9:**
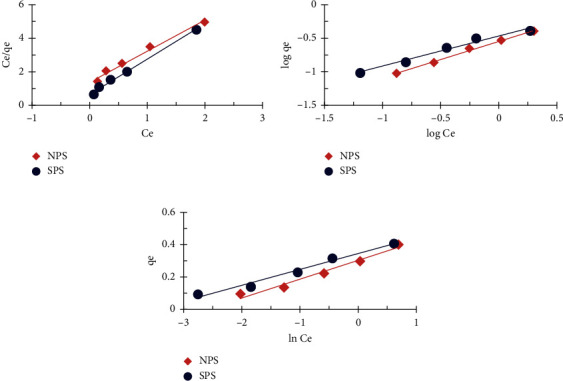
(a) Langmuir, (b) Freundlich, and (c) Temkin isotherm plots for the FD removal by NPS and SPS (1 g adsorbent dosage; 10 mg/L concentration of FD; 50 ml solution volume; 3 h contact time; and 25°C temperature).

Figure 9 and Table 2 presented a summary of isotherm model plots and parameters. NPS and SPS were well fitted with models from Langmuir, Freundlich and Temkin to remove the FD. The graphs display good correlation coefficient and linearity.

The maximum adsorption capacity of Langmuir, *q*_max_ (0.478 mg FD/g NPS and 0.542 mg FD/g SPS) [37], is higher than the experimental values (0.400 and 0.408 mg/g, respectively). It was calculated from the equation in which the favorable nature of adsorption was expressed:

(4) $$\begin{array}{c} R_{L}\;=\;\frac{1}{1+K_{L}C_{0}}\;. \end{array}$$

*R*_L_ values for FD removal by NPS and SPS were 0 < *R*_*L*_ < 1, indicating a favorable adsorption as shown in Table 2. *R*_*L*_ values generally indicate that the form of isotherm is irreversible (*R*_*L*_ = 0), favorable (0 < *R*_*L*_ < 1), linear (*R*_*L*_ = 1), or unfavorable (*R*_*L*_ > 1) [38].

Freundlich's isotherm model assumes that adsorption occurs on the adsorbent surface with the multilayer adsorption mechanism. KF values are 0.285 and 0.344 for NPS and SPS, respectively. The values of *n* were found in the range of *n* > 1, suggesting that the removal of FD by NPS and SPS is a desirable adsorption [38]. Similar results were obtained for previous studies to adsorb some basic dyes on untreated and treated beech sawdust samples [3]

The Temkin's isotherm model is concerned with adsorbent interactions with the FD. The values of (*A*) are 14.096 and 35.265 for NPS and SPS, respectively. The Temkin constant *B* was found to be 0.144 and 0.097 for NPS and SPS, respectively, which implies that the heat of adsorption of FD decreases with coverage due to adsorbate/adsorbate interactions [6, 39].

### 4.9. Adsorption Kinetic Model

The kinetic models were used to define the adsorption mechanism of FD by the adsorbents and to evaluate the dominant step in the reaction rate. The following models were expressed: pseudo-first-order (PFO), pseudo-second-order (PSO), liquid film diffusion (LFD), and intraparticle diffusion (IPD) [40].

(5) $$\begin{array}{c} \log\;\left(q_{e}-q_{t}\right)\;=\;\log\;q_{e}-\frac{K_{1}t}{2.303}\;,\\ \frac{t}{q_{t}}\;=\frac{1}{K_{2}q_{e}{}^{2}}\;+\;\frac{t}{q_{e}},\\ \ln\;\left(1-\;F\right)\;=-\;K_{\text{lfd}}\;t+C_{\text{lfd}},\\ q_{t}\;=\;K_{\text{ipd}}\;t^{\left(1/2\right)}\;-C_{\text{ipd}}, \end{array}$$

where *q*_*e*_ = the amount of FD adsorbed an equilibrium (mg/g), *q*_*t*_ = the amount of FD adsorbed at a time *t* (mg/g), *K*_1_ = the pseudo-first-order rate constant (g/mg.min), *K*_2_ = the pseudo-second-order rate constant (g/mg.min), *F* = *q*_*t*_/*q*_*e*_, *K*_*lfd*_ = the liquid film diffusion rate constant (1/min), t = the time (min), *C*_lfd_ = the boundary layer constant (mg g^−1^), *K*_ipd_ = the intraparticle diffusion rate constant (mg g^−1^.min^1/2^), and *C*_ipd_ = the constant of the boundary layer thickness (mg g^−1^).

**Figure 10:**
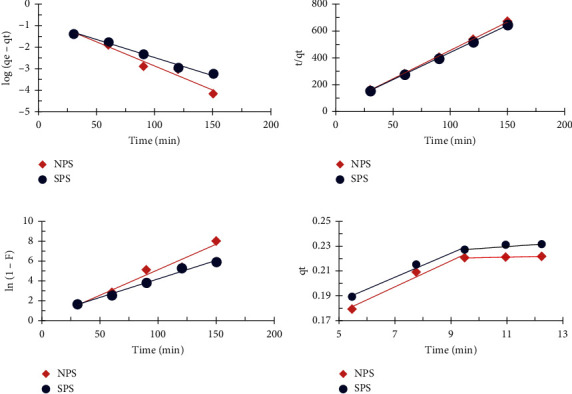
Plot of adsorption kinetic model (PFO, PSO, LFD, and IPD) for the FD removal by NPS and SPS (1 g adsorbent dosage; 10 mg/L concentration of FD; 50 ml solution volume; 3 h contact time; and 25°C temperature).

The linear plots of four kinetic models for removing FD by NPS and SPS are shown in Figure 10.  Table 3 described the parameters, constants, and coefficients of correlation (*R*^2^) of the four kinetic models. The experimental *q*_*e*_ values for dye were consistent with the calculated *q*_*e*_ values; therefore, the pseudo-first-order and pseudo-second-order models were well-fitted for the experimental data of the FD removal of NPS [40, 41]. Nevertheless, in the case of the removal of FD by SPS, the experimental *q*_*e*_ value for dye did not agree with the pseudo-first-order calculated *q*_*e*_ value, and it was not appropriate for the experimental data. In addition, the calculated *q*_*e*_ value (0.23) was very close to the experimental value (0.24) [41]; however, the pseudo-second-order fits well with the kinetic data. These results indicated that the rate of depigmentation was dependent on the rate at which active sites reached on the surface of the adsorbent, indicating the presence of a chemical process [42]. The correlation coefficients for the FD removal of the liquid membrane diffusion model (*R*^2^) are 0.960 and 0.986 by NPS and SPS, respectively, and are high, indicating that film diffusion is one of the factors determining the rate in the FD removal process. The values of liquid film diffusion (Klfd) show that removing FD with SPS (0.051) is faster than with NPS (0.037). The process consists of two steps, the first step being the full diffusion on the sawdust surface, and the second step being the gradual diffusion of the particles into the pores. It suggests that two or more steps were used to remove the dye [8, 30, 41].

### 4.10. Thermodynamics of Adsorption

Thermodynamic parameters, consisting of changes in entropy ∆S° (KJ/mol K°), enthalpy ∆H° (KJ/mol), and free-energy gibbs ∆G° (KJ/mol), are determined from the following equations [22]:

(6) $$\begin{array}{c} \ln\;K_{r}\;=\;\frac{\Delta S^{{}^{\circ}}}{R}-\;\frac{\Delta H^{{}^{\circ}}}{RT},\;\\ \Delta G^{{}^{\circ}}=-RT\;\ln\;K_{r},\\ K_{r}\;=\;\frac{C_{s}}{C_{e}},\; \end{array}$$

where *K*_r_ is the equilibrium constant, *C*_*s*_ and *C*_*e*_ (mg/L) are the dye concentrations on the adsorbent surface and in the equilibrium solution, *R* is the gas constant (8.314 mol/K), and *T* is the absolute solution temperature (K).

**Figure 11:**
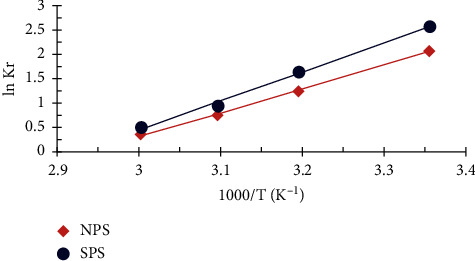
van't Hoff plots for the FD removal by NPS and SPS (1 g adsorbent dosage; 10 mg/L concentration of FD; 50 ml solution volume; 3 h contact time).

Figure 11 displays the plots for lnKr by van't Hoff against 1000/T and records the findings in Table 4. Negative values of *ΔH*° and *ΔS*° indicate that the adsorption process is exothermic and contributes to decreased entropy in solid/liquid interfaces during adsorption processes [6, 32]. The negative value of *ΔG*° shows that adsorption for the removal of FD is feasible and random. However, the values decreased with temperature rise from 298 K to 333 K, indicating a decline in the degree of spontaneity at high temperatures Generally, the negative values of ∆G° within −20 < ∆G° < 0 kJ/mol suggest that the physical reaction is the dominant mechanism [7, 32].

## 5. Conclusion

The results of the FIT-IR study revealed the existence of FD extraction groups of carboxyl, ethanol, alkanes, alkene, phenyl, and hydroxyl. The NPS and SPS surface SEM reveals the presence of many fine, deep pores that help remove a large quantity of FD molecules. The optimal results of FD removal were the use of 1 g of sawdust under standard conditions for a contact time of 120 min and pH 3. The results of the isotherm models used to describe the FD removal phenomenon on the sawdust surface showed a favorable removal for FD, and the Langmuir model is better than the Freundlich and Temkin models. Pseudo-first-order, second-order, liquid-film-diffusion, and intraparticle diffusion models were applied, confirming the elimination of FD by chemical adsorption and the internal diffusion process in two steps. The negative values of *∆H*° and *∆S*°, and *ΔG*° demonstrated the existence of spontaneous adsorption and the exothermic and lower randomness of the absorption. In addition, in eliminating the FD from aqueous solutions, SPS is better than NPS.

## Figures and Tables

**Table 1:** FT-IR peaks of the functional groups in NPS and SPS.

NPS, wavenumber (cm^−1^)	SPS, wavenumber (cm^−1^)	Frequency ranges (cm^−1^)	Groups (bonds)	Functional groups
3423	3415	3500–3200	O–H stretching	Phenolic or alcoholic
2915	2900	<3000	Sp^3^ C-H stretching	Alkane groups
1700	1690	1850–1600	C=O	Carbonyl group
1650	1650	1750–1600	C=C	Alkene groups
1450	1480	1500–1400	Sp^2^ C-H bending	Alkene groups
1340	1340	<1400	Sp^3^ C-H bending	Alkane groups
1250	1240	1300–1100	Sp^2^ C-O	Ester
1050	1050	1100–1000	Sp^3^ C–O	Alcohol
530	545	550–450	Sp^2^ C–H bending	Aromatic

**Table 2:** Adsorption isotherm parameters for the FD removal by NPS and SPS.

Adsorption isotherm models	Parameter	Adsorbent	
		NPS	SPS
Langmuir model	*q*_*m*_ (mg/g)	0.478	0.542
	*K*_*L*_ (L/mg)	3.053	1.304
	*R*_*L*_	0.061	0.133
	*R* ^*2*^	0.995	0.985

Freundlich model	*K*_*F*_ (mg^(1-1/n)^ g^−1^L^1/n^)	0.285	0.344
	*N*	1.828	2.235
	*R* ^2^	0.994	0.974

Temkin	*B* (J/mol)	0.114	0.097
	*A* (L/mg)	14.096	35.265
	*R* ^2^	0.975	0.979

**Table 3:** Parameters of adsorption kinetic model (PFO, PSO, LFD, and IPD) for the FD adsorption by NPS and SPS.

Kinetic models	Adsorbent	
	A (NPS)	B (SPS)
*q*_e,_, _exp_ mg g^−1^	0.222	0.2323
Pseudo-first order		
* q*_e, cal_ mg g^−1^	0.216	0.1391
* K*_1_, g/mg.min	0.051	0.0373
* R* ^2^	0.960	0.986
Pseudo-second order		
* q*_e, cal_ mg g^−1^	0.236	0.2463
* K*_2_, g/mg.min	0.532	0.4777
* h*, mg g^−1^.min	0.02953	0.02898
* t* ^½^	7.9778	8.4998
* R* ^2^	0.999	1
Liquid film diffusion		
* K*_lfd_, 1/min	0.0374	0.0512
* C*, mg g^−1^	0.5129	0.0272
* R* ^2^	0.986	0.960
Intraparticle diffusion		
* *Step 1		
* K*_ipd_, mg g^−1^.min^1/2^	0.010	0.095
* C*, mg g^−1^	0.124	0.139
* R* ^2^	0.968	0.985
* *Step 2		
* K*_ipd_, mg g^−1^.min^1/2^	0.0004	0.0016
* C*, mg g^−1^	0.217	0.213
* R* ^2^	0.908	0.860

**Table 4:** Thermodynamic parameters for the FD removal by NPS and SPS.

Adsorbent	Parameter			
	*T* (K)	∆*G*° (kJ/mol)	∆*H*° (kJ/mol)	∆*S*° (kJ/mol K)
NPS	298	−5.139	−40.677	−0.119
	313	−3.252		
	323	−2.010		
	333	−0.992		
	
SPS	298	−6.382	−49.583	−0.145
	313	−4.283		
	323	−2.571		
	333	−1.396		

## Data Availability

The data used to support the findings of this study are included within the article.
